# Serum 1,5-Anhydroglucitol to Glycated Albumin Ratio Can Help Early Distinguish Fulminant Type 1 Diabetes Mellitus from Newly Onset Type 1A Diabetes Mellitus

**DOI:** 10.1155/2020/1243630

**Published:** 2020-03-20

**Authors:** Lingwen Ying, Xiaojing Ma, Yun Shen, Jingyi Lu, Wei Lu, Wei Zhu, Yufei Wang, Yuqian Bao, Jian Zhou

**Affiliations:** Department of Endocrinology and Metabolism, Shanghai Clinical Center for Diabetes, Shanghai Diabetes Institute, Shanghai Key Laboratory of Diabetes Mellitus, Shanghai Jiao Tong University Affiliated Sixth People's Hospital, Shanghai 200233, China

## Abstract

**Background:**

Fulminant type 1 diabetes mellitus (FT1DM) onsets abruptly and usually occurs within 1 week after the onset of hyperglycemic symptoms. Glycated albumin (GA) and 1,5-anhydroglucitol (1,5-AG) are indicators that reflect short-term glucose levels. This study was aimed at investigating whether the 1,5-AG/GA index (AGI) is a suitable indicator for early FT1DM identification.

**Methods:**

A total of 226 subjects were enrolled, all with glycated hemoglobin A_1c_ (HbA_1c_) < 8.7%. FT1DM was diagnosed based on the 2012 Japan Diabetes Society criteria.

**Results:**

The AGI level was 0.54 (0.17–1.36) in the whole group. It was lower in FT1DM patients (0.16 [0.10–0.25]). Among the participants whose HbA_1c_ did not exceed 7.0%, the AGI of FT1DM decreased significantly compared to type 1A diabetes (T1ADM) and latent autoimmune diabetes in adults (LADA) patients (0.16 [0.12–0.26] *vs*. 0.46 [0.24–0.72] *vs*. 0.46 [0.24–0.72] *P* < 0.05). The receiver operating characteristic (ROC) curve showed that AGI can be used to distinguish FT1DM and T1ADM patients with HbA_1c_ < 8.7%. Diagnosing FT1DM based on AGI ≤ 0.3 only can help narrow down suspected FT1DM by up to 26.87%. If we diagnosed FT1DM when AGI was ≤0.3 and HbA_1c_ was ≤7.0%, the success rate further increased to 86.57%, among which 85.00% of FT1DM and 87.23% of T1ADM patients were successfully identified. Therefore, using the combination criteria of AGI and HbA_1c_ would improve the differential diagnosis efficacy by 61.11% compared with the AGI criterion only.

**Conclusion:**

AGI can help facilitate the early differential diagnosis of FT1DM and T1ADM when HbA_1c_ < 8.7%, with an optimal cut-off point of 0.3.

## 1. Introduction

Fulminant type 1 diabetes mellitus (FT1DM) is a new subtype of type 1 diabetes mellitus that is mainly characterized by an abrupt onset of a severe metabolic disorder, markedly elevated blood glucose level, normal or slightly elevated glycated hemoglobin A_1c_ (HbA_1c_), and almost complete or complete irreversible islet dysfunction [1, 2]. At present, FT1DM is mainly diagnosed based on the 2012 Japan Diabetes Society (JDS) criteria [3], specifically, based on the duration of symptoms, HbA_1c_, and C-peptide (both fasting and postload). However, HbA_1c_ has certain limitations since it mainly reflects the average blood glucose level of the past 2 to 3 months, which far exceeds the duration of FT1DM [4]. Moreover, the diagnosis of FT1DM is sometimes difficult to confirm, for two reasons: (1) because of its onset with diabetic ketosis (DK) or diabetic ketoacidosis (DKA), the patients are mostly in a fasting state, and the postload C-peptide cannot be obtained in a timely manner; (2) some type 1A diabetes (T1ADM) (i.e., classic type 1 diabetes) onsets with a HbA_1c_ less than 8.7%. Therefore, it is important to effectively identify FT1DM early, especially in patients with HbA_1c_ < 8.7%.

1,5-Anhydroglucitol (1,5-AG) has recently attracted extensive attention worldwide because it represents the average glucose of the past 1 to 2 weeks in diabetic patients and has some advantages in reflecting glucose fluctuations and postprandial hyperglycemia when compared with HbA_1c_ [5–7]. Several studies have shown that 1,5-AG is stable *in vivo* and decreases with increasing glucose level. Glycated albumin (GA), an indicator that reflects 2- to 3-week average glucose level, is recommended in stress hyperglycemia and has therapeutic effects evaluating after hypoglycated agent adjustment [8–10].

Therefore, we speculate that the 1,5-AG/GA index (AGI), combined with 1,5-AG and GA, can better compensate for the deficiency of HbA_1c_ in reflecting short-term glucose levels and help facilitate the differential diagnosis of FT1DM and T1ADM in patients whose HbA_1c_ < 8.7%. That is, the lower the AGI, the more likely FT1DM is. Therefore, the goal of the current study is to explore the AGI levels of subjects with different glucose metabolism statuses in the Chinese population and to clarify the identification efficacy and the optimal cut-off point of AGI for distinguishing FT1DM and T1ADM patients with HbA_1c_ < 8.7%.

## 2. Materials and Methods

### 2.1. Study Population

Twenty subjects who were first hospitalized and diagnosed with FT1DM (with their duration of symptoms ≤ 2 weeks and had complete clinical data and blood sample) in the Department of Endocrinology and Metabolism, Shanghai Jiao Tong University Affiliated Sixth People's Hospital, from January 2007 to July 2018 were recruited. Sex- and age-matched patients with newly diagnosed T1ADM (*n* = 47), type 2 diabetes mellitus patients (T2DM, *n* = 42) and nondiabetic participants (non-DM, *n* = 81; specifically, 33 with normal glucose tolerance and 48 with impaired glucose tolerance) through Propensity Score matching were included. In addition, 36 latent autoimmune diabetes in adults (LADA) patients with complete data were included. All subjects had an HbA_1c_ less than 8.7%. Finally, a total of 226 subjects were enrolled in this study.

This study was approved by the Ethics Committee of Shanghai Jiao Tong University Affiliated Sixth People's Hospital and followed the 1964 Declaration of Helsinki. Informed consent was obtained from all subjects enrolled in the current study.

### 2.2. Diagnostic Criteria

FT1DM was diagnosed according to the 2012 JDS criteria [3]: (1) DK or DKA occurred rapidly soon after the onset of hyperglycemic symptoms; (2) plasma glucose ≥ 16.0 mmol/L and HbA_1c_ < 8.7% (NGSP) at the first visit; and (3) fasting C-peptide  < 0.3 ng/mL (<0.10 nmol/L) and the peak value of C-peptide < 0.5 ng/mL (<0.17 nmol/L) after intravenous glucagon (or after meal) load at onset. Patients who met all the three of these criteria were diagnosed with FT1DM.

The glucose metabolism status determination and diabetes classification were determined based on 2010 American Diabetes Association (ADA) standards and the report of the expert committee on the diagnosis and classification of diabetes mellitus [11, 12].

### 2.3. Anthropometric and Biochemical Assessments

All subjects underwent a complete physical examination, including height, body weight, and blood pressure. Related information, including medical history, past history, and family history, was obtained. The body mass index (BMI) was defined as body weight/height^2^ (kg/m^2^). Laboratory data such as plasma glucose, HbA_1c_, GA, fasting C-peptide, and 2-hour C-peptide were collected. Serum 1,5-AG levels were measured by an enzymatic method (GlycoMark; GlycoMark Inc., New York, NY, USA) on a 7600 autoanalyzer (Hitachi, Tokyo, Japan) with intra-assay and interassay coefficients of variation (CVs) of <2.5% and <3.5%, respectively. GA was measured using an enzyme-based assay kit (Lucica GA-L, Asahi Kasei Pharma, Tokyo, Japan) on the 7600-120 automatic biochemistry analyzer (Hitachi, Tokyo, Japan). The intra-assay and interassay CVs were <3.5% and <5.0%, respectively. High-pressure liquid chromatography was used to quantify the levels of HbA_1c_ on a Variant II hemoglobin analyzer (Bio-Rad, Hercules, CA, USA). The other indexes were determined with standard methods [2].

### 2.4. Statistical Analysis

Data were analyzed with SPSS version 24.0 (SPSS, Inc., Chicago, IL, USA) and MedCalc version 15.2 (MedCalc Inc., Ostend, Belgium). All continuous variables are nonnormally distributed and presented as medians with interquartile ranges. Categorical variables are presented as percentages (%). The Mann-Whitney *U* test and the Kruskal-Wallis test were carried out for intergroup comparisons of nonnormally distributed variables, and the chi-squared test was used for intergroup comparisons of categorical variables. Sex- and age-matched patients were matched through the propensity score matching method. The receiver operating characteristic (ROC) curve was generated to analyze the value of related indicators in FT1DM identification. The optimal cut-off point was confirmed based on the Youden index. A *P* value of < 0.05 (two-tailed) was considered to be statistically significant.

## 3. Results

### 3.1. Clinical Characteristics of Study Participants

As shown in Table 1, a total of 226 subjects were enrolled in the current study, with 146 males and 80 females aged 41 (30–51) years old. The age of LADA patients was significantly higher than that of other groups (*P* < 0.01). Subjects with non-DM and T2DM had a significantly higher BMI than FT1DM, T1ADM, and LADA patients (all *P* < 0.01).

The average onset glucose level of FT1DM patients was 33.45 (27.03–40.76) mmol/L, with an HbA_1c_ of 6.5 (6.1–6.9)%, which was significantly higher than those of nondiabetic subjects but much lower than those of patients with T1ADM, LADA or T2DM (all *P* < 0.01). In addition, FT1DM patients suffered apparently impaired islet function (fasting C-peptide, 0.05 [0.01–0.18] ng/mL; 2-hour C-peptide, 0.11 [0.05–0.18] ng/mL).

### 3.2. Comparison of Related Glycemic Monitoring Indicators in Different Subgroups

**Figure 1:**
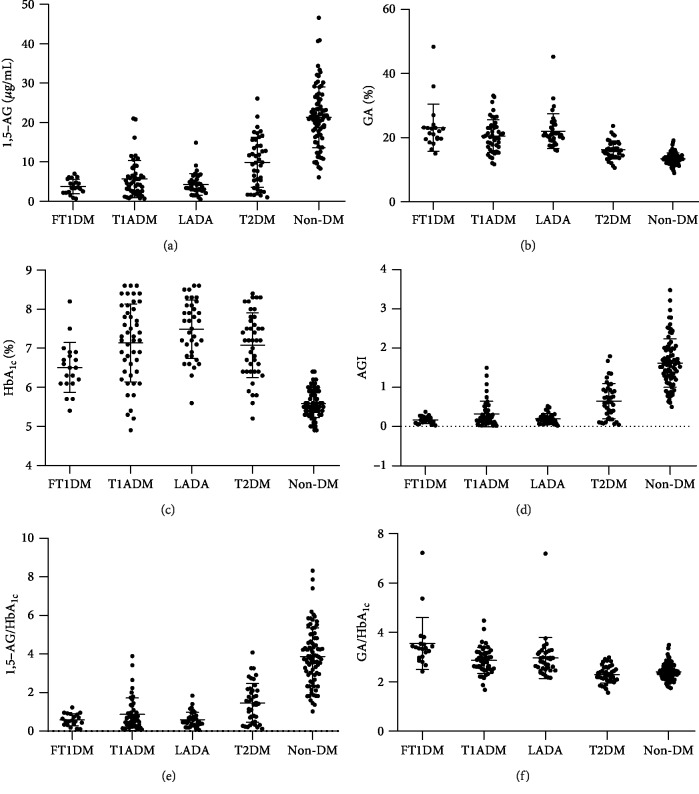
Related glycemic indicator levels ((a) 1,5-AG, (b) GA, (c) HbA_1c_, (d) AGI, (e) 1,5-AG/HbA_1c_, and (f) GA/HbA_1c_) in each subgroup with different glucose metabolic statuses. Abbreviations: 1,5-AG: 1,5-anhydroglucitol; AGI: 1,5-AG/GA index; FT1DM: fulminant type 1 diabetes mellitus; GA: glycated albumin; HbA_1c_: glycated hemoglobin A_1c_; LADA: latent autoimmune diabetes in adults; non-DM: nondiabetes mellitus; T1ADM: type 1A diabetes mellitus; T2DM: type 2 diabetes mellitus.

As shown in Figures 1(a)–1(c), serum 1,5-AG was highest in non-DM individuals, approximately 21.1 (16.5–25.5) *μ*g/mL, followed by T2DM (9.9 [3.9–14.5] *μ*g/mL), FT1DM (3.5 [2.3–5.5] *μ*g/mL), T1ADM (4.5 [2.2–7.0] ng/mL), and LADA (3.5 [2.8–5.6] ng/mL) (all *P* < 0.01). GA showed a completely opposite trend to 1,5-AG, and it was comparable between FT1DM, T1ADM, and LADA patients (21.7 [19.5–23.2]% *vs.* 20.2 [16.2–23.3]% *vs.* 20.8 [19.1–23.8]%, all *P* > 0.05), in whom it was significantly higher than that in T2DM (16.0 [14.0–18.3]%) and non-DM (13.4 [12.2–14.3]%) (all *P* < 0.01). However, the trend of GA was not exactly the same as that of HbA_1c_.

The AGI level was 0.54 (0.17–1.36) in the whole group. When compared between groups with different metabolism statuses, AGI was lowest in FT1DM patients (0.16 [0.10–0.25]), followed closely by T1ADM patients and LADA patients, and it was significantly higher in T2DM patients (0.62 [0.21–0.94]) and non-DM participants (1.57 [1.22–1.98]) (both *P* < 0.01). The trend of serum 1,5-AG and the 1,5-AG/HbA_1c_ ratio were the same as for AGI (Figures 1(d) and 1(e)). It should be emphasized that, among the participants whose HbA_1c_ did not exceed 7.0%, the AGI of patients with FT1DM was 0.16 (0.12–0.26), significantly lower than that of subjects with T1ADM, LADA, and T2DM and non-DM (0.46 [0.24–0.72], 0.31 [0.19–0.43], 0.77 [0.55–1.25], and 1.57 [1.22–1.98], respectively, all *P* < 0.05). However, the GA/HbA_1c_ ratio showed a different trend, being highest in FT1DM subjects, intermediate in T1ADM and LADA patients, and lowest in T2DM and non-DM subjects (Figure 1(f)).

### 3.3. AGI Can Help in the FT1DM Differential Diagnosis

**Figure 2:**
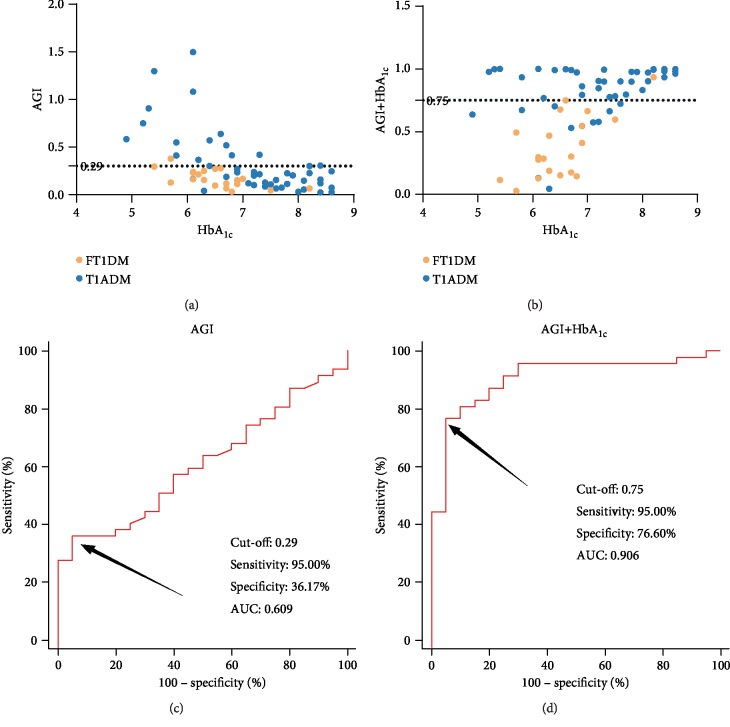
(a, c) AGI and (b, d) predictor of combined AGI with HbA_1c_ to differentiate FT1DM and T1ADM subjects with HbA_1c_ < 8.7%. Abbreviations: 1,5-AG: 1,5-anhydroglucitol; AGI: 1,5-AG/GA index; AUC: area under the curve; FT1DM: fulminant type 1 diabetes mellitus; GA: glycated albumin; HbA_1c_: glycated hemoglobin A_1c_; T1ADM: type 1A diabetes mellitus.

The ROC curve for the use of AGI in distinguishing FT1DM and T1ADM patients whose HbA_1c_ was <8.7% based on the 2012 JDS FT1DM diagnostic criteria are shown in Figure 2. The analysis demonstrated that the optimal AGI cut-off point for identification was 0.29, with a specificity of 36.17% (95% confidence interval [CI]: 22.67–51.48%), a sensitivity of 95.00% (95% CI: 75.13–99.87%), and an area under the curve (AUC) of 0.609 (95% CI: 0.482–0.726). Subsequently, we further investigated the efficacy of AGI combined with relevant glucose monitoring indicators in FT1DM identification. The results illustrated that the combination of AGI and HbA_1c_ may be the optimal solution for early differentiation of FT1DM and T1ADM in patients with HbA_1c_ < 8.7%, as it further increased the specificity to 76.60% (95% CI: 62.00–87.70%) and AUC to 0.906 (95% CI: 0.810–0.964) without affecting the sensitivity.

**Figure 3:**
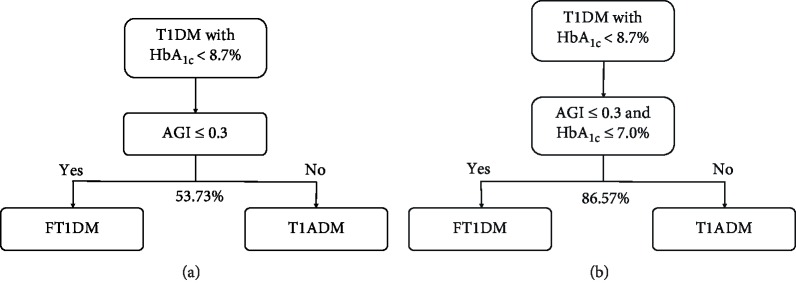
Strategies for application of glycemic indicators to differentiate FT1DM from T1ADM. (a) Differentiating FT1DM from T1ADM based on AGI only: AGI ≤ 0.3 to diagnose FT1DM and AGI > 0.3 to diagnose T1ADM can successfully distinguish 53.73% of participants if they all have HbA_1c_ < 8.7%, with suspected FT1DM narrowed down by 26.87%. (b) FT1DM was distinguished from T1DM (both FT1DM and T1ADM) with HbA_1c_ less than 8.7% based on the combined criteria of AGI and HbA_1c_: AGI ≤ 0.3 and HbA_1c_ ≤ 7.0% diagnosed FT1DM; otherwise, T1ADM. The identifying success rate became 86.57% under the combined criteria, increasing by 61.11% in comparison with the use of the AGI criterion only. Abbreviations: 1,5-AG: 1,5-anhydroglucitol; AGI: 1,5-AG/GA index; FT1DM: fulminant type 1 diabetes mellitus; GA: glycated albumin; HbA_1c_: glycated hemoglobin A_1c_; T1ADM: type 1A diabetes mellitus; T1DM: type 1 diabetes mellitus (including FT1DM and T1ADM).

For patients with HbA_1c_ < 8.7%, diagnosing FT1DM based on AGI ≤ 0.3 only successfully identified 53.73% of subjects, with 95.00% (19/20) of FT1DM patients screened out accurately, and narrowing down suspected FT1DM at a rate of 26.87% (Figure 3(a)). When we combined HbA_1c_ with AGI and diagnosed FT1DM when AGI ≤ 0.3 and HbA_1c_ ≤ 7.0%, the successful identification rate further increased to 86.57% (58/67), among which 85.00% (17/20) of FT1DM and 87.23% (41/47) T1ADM patients were successfully identified, with a positive predictive value (PPV) and negative predictive value (NPV) of 73.91% (95% CI: 51.59–89.77%) and 93.18% (95% CI: 81.34–98.57%), respectively. Therefore, the combined criteria of AGI and HbA_1c_ improved the differential diagnostic efficacy by 61.11% and further reduced the suspected FT1DM rate by 53.06% when compared with the AGI criterion only (Figure 3(b)).

## 4. Discussion

This is the first study to propose the new blood glucose monitoring parameter AGI. We noted that for individuals with newly diagnosed type 1 diabetes whose HbA_1c_ was less than 8.7%, the AGI criteria alone can help reduce the scope of the suspected FT1DM population by approximately 1/4, while the combined criteria of AGI and HbA_1c_ can successfully identify 5/6 patients, with an optimal cut-off point for AGI of 0.3.

FT1DM, a clinically critical illness, may result in serious complications, such as rhabdomyolysis, multiple organ failure, and acute renal failure [13–15], and may be diagnosed based on HbA_1c_, fasting C-peptide, and postload C-peptide. However, the postload C-peptide is often unavailable because of their fasting status at the time of the initial visit. Thus, it is of particular importance to seek a suitable indicator that can be tested in nonfasting status to distinguish FT1DM and T1ADM as early as possible, especially among those whose HbA_1c_ < 8.7%.

1,5-AG, a six-carbon monosaccharide, is stable *in vivo* and can be used for nonfasting detection [5, 16]. The reabsorption process of 1,5-AG can be competitively inhibited by glucose, which leads to a decline in serum 1,5-AG level under conditions of hyperglycemia, making 1,5-AG a short-term (1–2 weeks) glucose monitoring indicator [17, 18]. In addition, our recent study showed that the decrease in the AH index (formed by 1,5-AG combined with HbA_1c_) was an indication of recent intensified glucose metabolic disorders with poor islet *β* cell function [19]. GA is an indicator that mainly monitors the 2 to 3 weeks average glucose, has a good correlation with HbA_1c_, and is negatively correlated with serum 1,5-AG level. Consistent with our previous study [20], the indicator reflecting long-term glucose level (i.e., HbA_1c_) has not changed significantly, though higher than nondiabetic subjects, but much lower than other types of diabetic patients. However, in conformity with the extremely high onset plasma glucose level, the short-term indicator GA increased to 21.7% and 1,5-AG decreased to 3.5 *μ*g/mL, exceeding the cut-off value of GA (17.1%), which is used to detect diabetes in the Chinese population [21], and lower than 15.9 *μ*g/mL, which is the cut-off point to screen diabetes [22]. All these results are in accordance with the clinical features of abrupt onset and rapid development of FT1DM.

Moreover, we propose a new glucose monitoring parameter, AGI, that combines 1,5-AG with GA and may enhance the advantages of both indicators in reflecting short-term glucose level. We found that AGI was highest in non-DM individuals, second-highest by T2DM, and lowest in patients with FT1DM, T1ADM, and LADA. The trend of AGI in participants with different glucose metabolism statuses was consistent with serum 1,5-AG and opposite to the GA trend. HbA_1c_ was lowest in non-DM participants, followed by FT1DM, and highest in T1ADM, LADA, and T2DM patients. The phenomenon that the trends of HbA_1c_ and AGI were not exactly the same further verifies the characteristics of FT1DM of sudden onset and usually <1-week duration of symptoms.

We also noted for the first time that AGI can be used to distinguish T1ADM with HbA_1c_ < 8.7% and FT1DM, and the combination of AGI and HbA_1c_ performed the best for early identification. A study conducted by Koga et al. [23] enrolled 38 FT1DM and 31 acute-onset T1ADM patients and found that 8.7% was the optimal cut-off point of HbA_1c_ for identification. Additionally, whether GA exceeded 33.5% can be applied for differential diagnosis. A related study took 1,5-AG into consideration and included 7 FT1DM patients and 32 T2DM patients with HbA_1c_ less than 8.5% (JDS standard). The results demonstrated that 1,5-AG can be used to identify T2DM and FT1DM, but its sensitivity is slightly lower than that of GA [4]. Another study, also conducted by Koga et al. [24] in the same period, included 35 FT1DM patients and 42 T2DM participants whose HbA_1c_ was <8.5% (JDS standard), and the results indicated that GA/HbA_1c_ > 3.2 should be the best distinguishing indicator. In contrast to all three studies mentioned above, this study focused on T1ADM with HbA_1c_ < 8.7% and FT1DM. Our results demonstrated that by diagnosing FT1DM when AGI ≤ 0.3, 95.00% of FT1DM can be identified successfully, and the suspected FT1DM group was narrowed down by more than 1/4.

Based on FT1DM and acute-onset type 1 diabetes individuals, the JDS results showed that 70.2% of participants had an onset HbA_1c_ < 7.0%, and the average onset HbA_1c_ in patients with FT1DM was 6.8%. The specificity of FT1DM diagnoses can reach 100.0% if the set cut-off point of HbA_1c_ (NGSP) ≤ 8.5% [3]. In the current study, the overall average of HbA_1c_ was similar to 6.4 ± 0.9% of the Japanese patient that reported previously [25], and the FT1DM patients showed a significant decline of AGI compared with T1ADM patients in the HbA_1c_ ≤ 7.0% subgroup. Based on the results mentioned above, we found that if we added HbA_1c_ to AGI, then diagnosing FT1DM at AGI ≤ 0.3 and HbA_1c_ ≤ 7.0% accurately distinguished 86.57% of individuals, with its differential diagnostic efficacy of combined criteria increasing by 61.11% when compared with AGI only, and providing a basis for early identification and therapy.

The current study enrolled participants with non-DM, FT1DM, T1ADM, LADA, and T2DM simultaneously and can better define the clinical features of diabetic and non-DM subjects as well as different subtypes of diabetes patients to provide evidence for clinical diagnosis and therapy. However, there are still some limitations. First, the sample size was relatively small because of the low incidence of FT1DM. Second, this was a cross-sectional study, so further prospective studies are needed to verify the efficacy of AGI and HbA_1c_ combined criteria in the differential diagnosis of FT1DM and T1ADM with HbA_1c_ < 8.7%.

In summary, this study proposed the glucose monitoring parameter AGI for the first time and found that AGI can be used for the early differential diagnosis of FT1DM and T1ADM with HbA_1c_ < 8.7%, with an optimal cut-off point for AGI of 0.3.

## Figures and Tables

**Table 1:** Clinical characteristics of the study participants.

	FT1DM (*n* = 20)	T1ADM (*n* = 47)	LADA (*n* = 36)	T2DM (*n* = 42)	Non-DM (*n* = 81)
Male, *n* (%)	16 (80.00)	27 (57.45)	19 (52.78)^#^	32 (76.19)	52 (64.20)
Age (year)	32 (28–44)	38 (27–47)	65 (57–69)^##^	44 (30–48)	36 (31–45)
BMI (kg/m^2^)	22.48 (20.25–23.66)	22.21 (20.81–23.94)	23.56 (21.57–25.51)	25.84 (23.71–30.51)^††^	25.39 (22.26–27.76)^‡‡^
SBP (mmHg)	110 (105–120)	120 (110–130)^∗∗^	130 (119–135)^##^	130 (120–141)^††^	123 (114–136)^‡‡^
DBP (mmHg)	70 (60–80)	75 (70–80)^∗^	79 (70–80)^#^	80 (72–90)^††^	77 (71–83)^‡‡^
DM family history, *n* (%)	5 (25.00)	13 (27.66)	18 (50.00)	25 (59.52)^†^	31 (38.27)
1,5-AG (*μ*g/mL)	3.5 (2.3–5.5)	4.5 (2.2–7.0)	3.5 (2.8–5.6)	9.9 (3.9–14.5)^††^	21.1 (16.5–25.5)^‡‡^
GA (%)	21.7 (19.5–23.2)	20.2 (16.2–23.3)	20.8 (19.1–23.8)	16.0 (14.0–18.3)^††^	13.4 (12.2–14.3)^‡‡^
HbA_1c_ (%)	6.5 (6.1–6.9)	7.2 (6.4–8.0)^∗∗^	7.6 (6.8–8.1)^##^	7.2 (6.4–7.7)^††^	5.5 (5.4–5.9)^‡‡^
AGI	0.16 (0.10–0.25)	0.21 (0.11–0.41)	0.18 (0.09–0.27)	0.62 (0.21–0.94)^††^	1.57 (1.22–1.98)^‡‡^
FCP (ng/mL)	0.05 (0.01–0.18)	0.22 (0.02–0.61)^∗^	0.66 (0.03–1.37)^##^	2.21 (1.80–3.25)^††^	2.20 (1.67–2.78)^‡‡^
2hCP (ng/mL)	0.11 (0.05–0.18)	0.22 (0.02–1.27)	0.92 (0.02–3.77)^#^	6.51 (4.58–8.72)^††^	10.22 (8.17–13.09)^‡‡^

Data were expressed as median (interquartile range) or *n* (%). Abbreviations: 1,5-AG: 1,5-anhydroglucitol; 2hCP: 2-hour C-peptide; AGI: 1,5-AG/GA index; BMI: body mass index; DBP: diastolic blood pressure; FCP: fasting C-peptide; FT1DM: fulminant type 1 diabetes mellitus; GA: glycated albumin; HbA_1c_: glycated hemoglobin A_1c_; LADA: latent autoimmune diabetes in adults; NDM: nondiabetes mellitus; SBP: systolic blood pressure; T1ADM: type 1A diabetes mellitus; T2DM: type 2 diabetes mellitus. ^∗^*P* < 0.05 and ^∗∗^*P* < 0.01, FT1DM *vs*. T1ADM. ^#^*P* < 0.05 and ^##^*P* < 0.01, FT1DM *vs*. LADA. ^†^*P* < 0.05 and ^††^*P* < 0.01, FT1DM *vs*. T2DM. ^‡^*P* < 0.05 and ^‡‡^*P* < 0.01, FT1DM *vs.* non-DM.

## Data Availability

The data that support the findings of this study are available from the corresponding author upon reasonable request.

## Abbreviations

1,5-AG: 1,5-Anhydroglucitol
ADA: American Diabetes Association
AGI: 1,5-AG/GA index
AUC: Area under the curve
BMI: Body mass index
CI: Confidence interval
CV: Coefficients of variation
DK: Diabetic ketosis
DKA: Diabetic ketoacidosis
FT1DM: Fulminant type 1 diabetes mellitus
GA: Glycated albumin
GADA: Glutamic acid decarboxylase
HbA_1c_: Glycated hemoglobin A_1c_
IA_2_-Ab: Islet-associated antigen 2
JDS: Japan Diabetes Society
LADA: Latent autoimmune diabetes in adults
Non-DM: Nondiabetic participants
NPV: Negative predictive value
PPV: Positive predictive value
ROC: Receiver operating characteristic
T1ADM: Type 1A diabetes mellitus
T2DM: Type 2 diabetes mellitus patients.
